# IL-33 and the Cytokine Storm in COVID-19: From a Potential Immunological Relationship towards Precision Medicine

**DOI:** 10.3390/ijms232314532

**Published:** 2022-11-22

**Authors:** Fabiana Furci, Giuseppe Murdaca, Alessandro Allegra, Luca Gammeri, Gianenrico Senna, Sebastiano Gangemi

**Affiliations:** 1 Asthma Centre and Allergy Unit, University of Verona and Verona University Hospital, 37124 Verona, Italy; 2 Department of Clinical and Experimental Medicine, School and Operative Unit of Allergy and Clinical Immunology, Policlinico G. Martino, University of Messina, 98100 Messina, Italy; 3 Department of Internal Medicine, University of Genoa, 16126 Genoa, Italy; 4 Division of Hematology, Department of Human Pathology in Adulthood and Childhood “Gaetano Barresi”, University of Messina, 98122 Messina, Italy; 5 Department of Medicine, University of Verona and Verona University Hospital, 37124 Verona, Italy

**Keywords:** IL-33, COVID-19, cytokine storm, inflammation, therapeutic strategies

## Abstract

Coronavirus SARS-CoV-2 has represented, and still represents, a real challenge from a clinical, diagnostic and therapeutic point of view. During acute infection, the increased levels of pro-inflammatory cytokines, which are involved in the pathology of disease and the development of SARS-CoV-2-induced acute respiratory disease syndrome, the life-threatening form of this infection, are correlated with patient survival and disease severity. IL-33, a key cytokine involved in both innate and adaptive immune responses in mucosal organs, can increase airway inflammation, mucus secretion and Th2 cytokine synthesis in the lungs, following respiratory infections. Similar to cases of exposure to known respiratory virus infections, exposure to SARS-CoV-2 induces the expression of IL-33, correlating with T-cell activation and lung disease severity. In this work, we analyse current evidence regarding the immunological role of IL-33 in patients affected by COVID-19, to evaluate not only the clinical impact correlated to its production but also to identify possible future immunological therapies that can block the most expressed inflammatory molecules, preventing worsening of the disease and saving patient lives.

## 1. Introduction

SARS-CoV-2, a single-stranded RNA virus responsible for the coronavirus disease 2019 (COVID-19), first reported in China in December 2019, can be considered the cause of “the twenty-first century disease” [1]. COVID-19 is characterized by various clinical presentations, from asymptomatic cases to multi-organ disease [2]. The pathophysiology of COVID-19, incompletely understood, is related to a dysregulated response of the immune system, characterised by uncontrolled production of pro-inflammatory cytokines, such as interleukin (IL)-1β, IL-6, IL-8 and IL-17 and tumour necrosis factor (TNF α), by immune and non-immune effector cells [3]. The release of these cytokines contributes to the symptoms and severity of the disease, and activation of the complement system is associated with this [4,5,6].

Among the determinants of disease severity, age plays a key role; indeed, the majority of infected young individuals presented mild disease, not requiring hospitalization [7]. However, despite this evidence, one cannot fail to consider that multisystem inflammatory syndrome in children (MIS-C), a specific paediatric complication of SARS-CoV-2 infection, presents a pathophysiology of MIS-C that is largely unknown, but which is generally attributed to the cytokine storm reported in critically diseased adults. In addition, young individuals who present mild COVID-19 disease seem to have the same risk as hospitalized patients for developing persistent fatigue, dyspnoea, “brain fog” and other symptoms often known as a post-COVID syndrome [8].

Another important determinant of disease severity is sex, finding that men are over-represented among patients with severe acute disease, probably due to differences in the elicited immune responses, whereas women have a high risk for developing post-COVID syndrome [9].

The major event underlying the progression of the disease is a cytokine storm. The underlying mechanisms responsible for this unconstrained inflammatory cytokine storm are not yet well understood, but there are several hypotheses. The first hypothesis is based on virus replication, which induces pyroptosis, a highly inflammatory form of lytic-programmed cell death (apoptosis). In COVID-19 patients, this form of apoptosis causes the release of pro-inflammatory cytokines and alters the function of macrophages and lymphocytes, inducing peripheral lymphopenia [10,11,12]. Much available evidence concerns an alteration in innate immunity induced by type 1 IFN [10,13]. Virus infection, macrophages, dendritic cells and neutrophils are responsible for the activation of the immune response as the body’s first-line defence. According to this mechanism, the study of lung autopsies from patients who died from COVID-19 reported a high infiltration of macrophages in the bronchial mucosa [14]. As reported above, many studies have highlighted an increased production of some cytokines, such as IL-6, in patients affected by COVID-19 [15]. Indeed, a second hypothesis is based on the activation of adaptive immunity and on the production of neutralizing antibodies against the surface antigens of the virus, which are related to the onset of severe respiratory disease in COVID-19 patients. In the literature, it has been reported that immunoglobulin (Ig) Gs could bind to the S protein, activating inflammatory cascades and resulting in an accumulation of pro-inflammatory macrophages and monocytes in the lungs through the release of IL-8 and monocyte chemoattractant protein (MCP)-1. The inflammatory reaction is mediated by Fc receptor (FcR) interaction on the surface of monocytes/macrophages with the virus-anti-S-IgG complex. This notion is supported by the decreased level of pro-inflammatory cytokine following the blockage of macrophage receptors [12,13]. Therefore, the study of cytokine storms could play a relevant role in the evaluation of patients affected by COVID-19 to understand any possible link with disease severity, and highlights the importance of management of cytokine release syndrome (CRS) as an interesting approach to COVID-19 therapy.

## 2. The Biology of IL-33

IL-33 is a 270-amino-acid protein, and a member of the IL-1 family. It is a cytokine that plays a key role in tissue homeostasis and repair, type 2 immunity, allergic and non-allergic inflammation, viral infection and cancer. It is a member of the “alarmins” family, released in response to cellular damage, apoptosis or immune activation. Alarmins play a key role as intercellular signals by interacting with chemotactic and pattern recognition receptors (PRRs) to boost immune cells in the host’s defence response. Taking into account their ability to activate dendritic cells (DCs) in their mature form, alarmins act in association with adaptive immunity and T-cell-dependent long-term immune memory [16].

Released after cellular damage or tissue injury, IL-33 is found in the nuclei of tissue-derived cells, in endothelial cells from blood vessels, in epithelial cells from barrier tissues and in fibroblastic stromal cells. When IL-33 is released, it induces the activation of MyD88-dependent signalling pathways in cells that express the ST2 (IL-1RL1) receptor [17,18].

The expression of ST2 is constitutively expressed in other immune cells, such as NK, iNKT cells and neutrophils. In other cell types, expression of the IL-33 receptor is inducible and is related to the tissue microenvironment. This aspect is represented, for example, by the action of IL-12 in inducing the ST2 receptor on Th1 cells and CD8 T cells, and IL-33 is a crucial cytokine for the activation of these cells [19,20,21]. IL-33 can act both intracellularly as a nuclear factor able to regulate gene expression and extracellularly as an IL-1 family cytokine. Its ability to act as an extracellular receptor that activates immune cells is mainly related to its structure [16]. The full-length human IL-33 protein is composed of an N-terminal nuclear domain, which is fundamental for bringing the cytokine into the nucleus, and a C-terminal IL-1-like cytokine domain [22,23,24]. The two domains are separated by a divergent “protease sensor” domain. The C-terminal domain (cytokine domain, aa 112–270) possesses cytokine activity and has a three-dimensional structure similar to interleukin 1 (IL-1), whereas the ”protease sensor” domain is a cleavage platform for many proteases [25,26,27,28]. It should be noted that there are many species-specific differences among IL-33 levels in various tissues: expression is constitutive in mouse keratinocytes while it is inducible in human keratinocytes [29,30]. Most IL-33 production in the murine lung is derived from alveolar type II pneumocytes (ATII), while in human lungs most production of IL-33 is from airway basal epithelial cells and endothelial cells [31,32,33]. Moreover, although IL-33 levels are abundant in the alveoli of both species, cellular sources are not the same; cytokine derivation is from ATII epithelial cells in mice and from alveolar endothelial cells in humans [33,34].

The IL-33/ST2/IL1RAcP is responsible for dimerization of the toll-interleukin receptor (TIR) domain; it induces the activation of intracellular signalling through the myeloid differentiation primary response 88 (MyD88) adaptor, interleukin receptor-associated kinase (IRAK)1, IRAK4 kinases and tumour-necrosis-factor-receptor-associated factor (TRAF)6. All these mechanisms lead to the consequent activation of mitogen-activated protein (MAP) kinases and nuclear factor κB (NFκB) transcription factors, inducing the pro-inflammatory cascade. Moreover, this complex is also responsible for the activation of Jun kinase and extracellular-signal-regulated kinase (ERK) expression, which induces the down-regulation of forkhead box p3 (Foxp3) and GATA3 expression. The prevalent expression of ST2 by mast cells, group 2 innate lymphoid cells (ILC2s), eosinophils and regulatory T cells (Tregs) allows us to understand how these cells represent the major target of IL-33, alarmin, with a key role in modulating immune cell function in many conditions such as asthma and lung diseases [16,35].

## 3. The Role of IL-33 Signalling in COVID-19 Inflammatory Status

**Figure 1:**
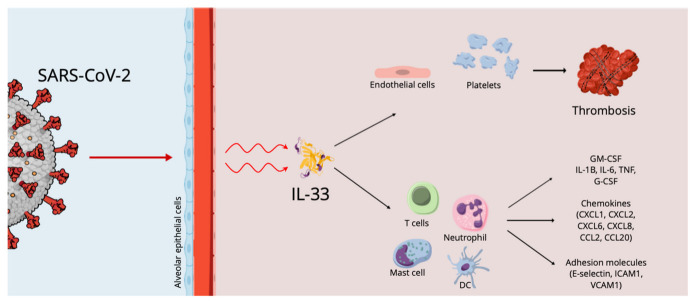
The effects of SARS-CoV-2 on the alveolar epithelium.

The involvement of various molecules, cytokines and immune cells has been described in COVID-19 (Figure 1), but a key concept to highlight is that the cells damaged by SARS-CoV-2 release alarmins, or damage-associated molecular patterns (DAMPs), which act as danger molecules in promoting inflammatory response [36,37]. IL-33, as reported above, is recognized as an alarmin, an expression of cellular damage or infection, whose increased levels in epithelial and endothelial cells recall its pro-inflammatory role in respiratory diseases. In particular, the mature bioactive form of IL-33 requires cleavage by proteases. In the literature, it has been reported that serum IL-33 levels are up-regulated in elderly patients affected by COVID-19, an expression of the epithelial damage induced by the interaction between the airway epithelium and activated immune cells, linking them to severe outcomes [38]. These data imply a key role for IL-33 in COVID-19 pathogenesis. However, SARS-CoV-2-derived papain-like protease (PLpro), an inducer of IL-33 in epithelial cells, could also trigger epithelium-derived IL-33, inducing inflammation in the lungs.

It is equally important to highlight that IL-33 can stimulate antiviral cytotoxic T-cell action and the production of antibodies. Stanczak MA. et al., further highlighting the role of IL-33 in COVID-19 immunobiology, reported that the persistent production of IL-33 in response to T-cell activation may be useful in the case of later contact with the virus [39].

Liang et al. infected two human epithelial cell lines, Fadu and LS513, with SARS-CoV-2 in vitro, reporting a significant increase in IL-33 transcript levels in both cell lines at 72 h post-infection [38].

Marcovic SS. et al. analysed the correlation of IL-33 and other innate immunity cytokines with COVID-19 severity in patients with COVID-19 that were divided into two groups (mild/moderate and severe/critical). The authors reported that in a more progressive stage of the disease, increased IL-33 levels are a determinant in lung inflammation favouring the production of innate pro-inflammatory cytokines such as IL-1β, IL-6, TNF-α, IL-12 and IL-23 in several target cells, causing the most severe forms of COVID-19. The authors also reported a positive correlation between IL-33 and clinical parameters of COVID-19, in particular higher values of the neutrophil count, C-reactive protein (CRP), D dimer, procalcitonin (PCT), lactate dehydrogenase (LDH), urea, creatinine, creatine kinase CK, ferritin, total bilirubin (TBIL) and aspartate aminotransferase (AST), as well as lower values of lymphocyte and monocyte count and albumin. Serum levels > 332.08 pg/mL of IL-33 were considered a factor related to the risk of increased severity of COVID-19 [40]. The same results were reported by Wang et al. and by Rubio-Sánchez et al. [41,42].

Meidaninikjeh S. et al. and Miyazawa M. reported that cellular compositions of lung infiltrates in patients with COVID-19 pneumonia are different depending on the progression of the disease. In detail, in patients with moderate pneumonia, infiltrates are made up mainly of lymphoid and dendritic cells, while in patients with the severe form of the disease there is a massive infiltration of macrophages and neutrophils [43,44]. IL-33 induces neutrophil migration via macrophage-derived CXCL1 and CXCL2, whereas neutrophil elastase and cathepsin G are responsible for IL-33 processing and maturation, which leads to inflammatory responses. In agreement with these data, Marcovic SS. et al. highlighted an increased neutrophil count in patients with severe COVID-19 [40].

Focusing on seroconversion, in the literature, it has been reported that IL-33 clustered together with anti-receptor binding domain (RBD) IgG in moderate and severe cases [45]. Stanczak M.A. et al. reported that the SARS-CoV-2-infection-induced production of IL-33 clustered together with IgG titres and that after recovery from COVID-19 patients still had persistent circulating PBMCs that produced IL-33 in response to virus-specific T-cell activation, correlating with seropositivity [39]. Zeng G. et al. studied soluble ST2 (sST2, a cardiac biomarker) levels among COVID-19 patients and a possible relationship between inflammatory status and disease severity, highlighting a positive correlation between serum sST2 levels and CRP, and a negative correlation between CD4+ and CD8+ T lymphocyte counts and sST2. In particular, Zeng Z. et al. analysed the relationship of serum sST2 to lymphocyte subsets, reporting that CD3+CD4+ and CD3+CD8+ lymphocyte absolute counts were negatively correlated with the levels of serum sST2 in patients with COVID-19, highlighting that the elevated serum sST2 may favour the dysfunction of T cells in COVID-19 progression [46]. This concurs with many studies reporting that during COVID-19 infection patients are often characterized by low lymphocyte counts and impaired cytotoxic activity [47]. Gaurav et al. compared lung sections of COVID-19 patients to those of normal patients and patients with chronic inflammatory lung diseases, such as IPF and COPD, highlighting that tissue IL-33 levels were higher among the latter. Indeed, patients with COVID-19 had very low IL-33 expression, which was significantly reduced compared to that of control subjects. IL-33 levels were increased in post-COVID fibrosis and were higher compared to the levels in patients affected by COPD and IPF [48].

### Cytokine Storm, IL-33 Effects and Thrombosis in COVID 19 Infection

The most frequent complication of systemic infections is the triggering of the coagulation process, which can exhibit a wide variety of clinical symptoms fluctuating from subclinical expression, represented by increased laboratory indicators for fibrin and thrombin products, to the appearance of thrombosis and disseminated intravascular coagulation [49].

**Figure 2:**
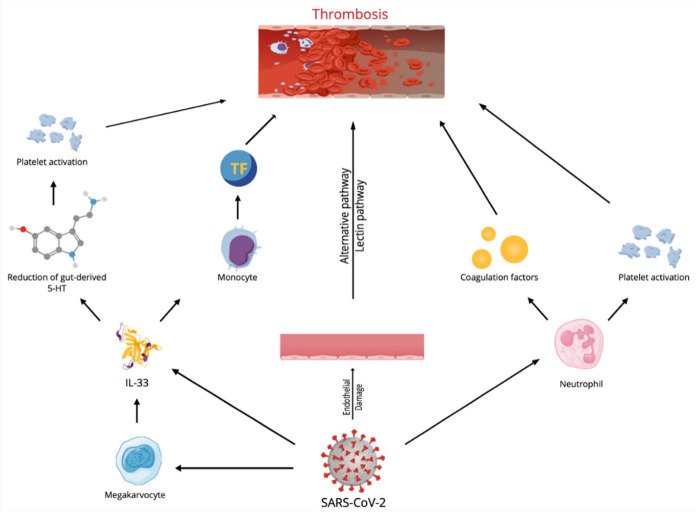
Mechanisms responsible for immunothrombosis in SARS-CoV-2 infection. TF = tissue factor.

Regarding COVID-19 patients, the devastating occurrence of uncontrolled coagulopathy is a powerful prognosticator of mortality in infected subjects [50]. However, the genesis of hypercoagulable condition and thrombosis correlated to COVID-19 is uncertain [51,52,53]. COVID-19 infection likely provokes a condition of immune system hyperstimulation, which has been defined as immunothrombosis (Figure 2), in which stimulated neutrophils react with activated platelets and the coagulation factors, causing intravascular clot generation in all vessels. It is supposed that the overstated immunothrombosis that occurs within pulmonary microvessels is the leading cause of COVID-19 symptoms [54,55].

A different motive for stimulating the coagulation cascade is represented by endothelial cell damage and activation by SARS-CoV-2 infection itself. Assessment of skin and pulmonary autopsies of subjects who died of COVID-19 infection demonstrated the presence of thrombosis and microvascular damage, coherent with severe, widespread stimulation of alternative and lectin-founded paths of the complement system [56] and successive stimulation of the clotting cascade, provoking fibrin accumulation [57]. The resulting hypercoagulable condition is the main reason for venous thromboembolism, pulmonary embolism and deep venous thrombosis of the lower extremities, which has been reported in subjects affected by COVID-19.

Recently, an in vitro experiment confirmed the role of the cytokine storm in the generation of thrombosis in COVID-19 patients [58], and IL-33 plays an essential role in the cytokine storm, especially regarding the triggering of pro-thrombotic phenomena.

Indeed, IL-33 stimulates endothelial cells (ECs), causing the onset of an inflammatory phenotype via an increase in different molecules such as intercellular adhesion molecule-1, vascular cell adhesion molecule-1, monocyte chemoattractant protein-1 and endothelial-selectin or cytokines such as IL-6 and Il-8 [59,60]. IL-33 also supports angiogenesis and regulates the proteolytic ability of ECs by easing the generation of plasminogen activator inhibitor type-1 and urokinase-type plasminogen activator [61,62]. In a different patient setting, several reports showed that IL-33 serum concentrations are correlated with reduced survival in subjects with ST elevation myocardial infarction [63,64], while an experiment demonstrated that increased levels of IL-33 after coronary stent implantation are correlated with coronary restenosis [65].

Furthermore, tissue factor (TF) is the main factor of blood coagulation and has a critical effect in the onset of thrombotic events. IL-33 stimulated TF mRNA and protein in umbilical and coronary artery ECs in an ST2- and NF-κB-dependent modality, but this increase was IL-1-independent. IL-33-exposed ECs decreased the coagulation time of plasma and whole blood, while in atherosclerotic plaques, TF mRNA was related to IL-33 mRNA generation. Moreover, IL-33 and TF protein were found in the same location of clot generation in plaques of subjects with carotid stenosis. Thus, IL-33 could increase thrombotic ability and operate on thrombus formation via the production of TF in ECs [66].

However, the effects of IL-33 on TF production could also occur via other mechanisms. Monocytes and monocyte-derived microvesicles (MVs) are one of the principal origins of TF. Increased monocyte-derived TF and an increased circulating amount of procoagulant MVs participate in the establishment of a prothrombotic state. IL-33 was reported to induce a time- and dose-dependent increase in monocyte TF concentrations. IL-33-exposed monocytes also released CD14+TF+ MVs and IL-33 was reported to increase the TF action of monocytes and monocyte-originated MVs. Intermediate monocytes (IMs) displayed the greatest receptor expression, with a reduced expression on non-classical monocytes (NCMs) and classical monocytes (CMs). IL-33 caused a relevant increase in TF only in the IM, while a reduced expression was reported in NCMs, and no increase in CMs was noted. Thus, IL-33 may further participate in the establishment of a prothrombotic condition [67].

Beside the potent effect on TF generation, in vitro experiments demonstrated IL-33 was able to reduce the production of TF pathway inhibitor in ECs. This effect influences the coagulation time of whole blood and plasma ex vivo [14]. Furthermore, IL-33-caused generation of adhesion molecules on ECs could cause an increased binding and stimulation of white cells and leukocyte-originated MVs to ECs [68,69,70].

Furthermore, the strict, bidirectional relationships between IL-33 and platelets and their precursors are of particular significance. A study performed on an animal experimental model of intestinal inflammation displayed the precise effect of IL-33 in stimulating platelets. Inflammation increases systemic platelet triggering and blood coagulation, generating increased concentrations of IL-33 and provoking platelet activation through a rise in 5-HT release. More relevantly, authors assessed that deficiency of epithelial-originated IL-33 reduced 5-HT concentration, causing an altered platelet activation [71].

Finally, though platelets are the most relevant effectors of thrombosis, they are also immune cells. Platelets interact with immune cells, modifying their activity, maturation and proliferation, interfering with all expression of the immune response. Megakaryocytes (Mks) are the bone marrow precursors of circulating platelets, and until lately Mks were only believed to be bone-marrow-occupant cells. However, Mks also are present in the lung, and these Mks present higher numbers of immune molecules than bone marrow Mks. A study attempted to assess the immune activities of lung Mks (MkLs). They reported that MkLs had gene expression profiles that are analogous to antigen-presenting cells, and the immune phenotype was variable and regulated by the tissue immune milieu, as demonstrated by bone marrow Mks presenting an MkL-like phenotype after exposure to immune molecules, such as IL-33. In vitro and in vivo experiments showed that MkL assumed and managed antigenic proteins and pathogens, stimulated CD4+T cell triggering in an MHC II-dependent modality [72] and may have a central role in IL-33-induced thrombosis in COVID 19 patients.

A direct or indirect intervention aimed at modulating the effects of the IL-33 on the homeostasis of the coagulative system could guarantee an effective prophylactic or therapeutic action in the catastrophic thrombosis induced by the cytokine storm that complicates the course of COVID-19 infection.

## 4. Discussion and Prospects

COVID-19 has caused millions of deaths since its emergence in 2019 due to uncontrolled inflammation, dysregulated immune response, cytokine storms and an imbalanced hyperactive immune system. The cytokine storm causes multiple organ failure and lung immunopathology. The number of deaths and patients requiring intensive care has been a critical point in the epidemic, inducing the study of the pathophysiology underlying severe COVID-19 disease [73].

The identification of cytokines/chemokines and the pathophysiological features of COVID-19-induced cytokine storms is relevant in order to predict clinical deterioration, any requirement for hospitalization, or intubation or mortality. Moreover, this information is not only clinically but also therapeutically relevant, in that it may lead to the development of effective treatments [74]. The SARS-CoV-2 infection acts on cells by attaching itself to the angiotensin-converting enzyme 2 (ACE2) and/or type II transmembrane serine protease (TMPRSS2) receptors [75]. Subsequent viral replication and release is responsible for cellular damage with the release of unleashed pathogen-associated molecular patterns (PAMPs). In the early days of infection, the recognition of PAMPs by cells, such as macrophages, dendritic cells (DCs), neutrophils and natural killer (NK) cells, triggers the production of several pro-inflammatory cytokines and chemokines, activating innate immunity [76]. Therefore, while immunosuppression is conveyed through immunosuppressive cytokines such as transforming growth factor beta (TGFβ) and IL-10, the dysregulated immune response is induced by the rapid production and release of pro-inflammatory cytokines, such as cytokines of the IL-1 family, TNFα and IL-6 [77,78]. In a more progressive stage of the disease, the continued injury of alveolar epithelial cells caused by interactions between the airway epithelium and activated immune cells is followed by an overproduction of IL-33, promoting lung inflammation through the production of many innate pro-inflammatory cytokines, such as IL-1 β, IL-6, TNF-α, IL-12 and IL-23 in several target cells, causing the most severe forms of COVID-19 [40].

Therefore, the release of IL-33, a member of the IL-1 family, which induces an inflammatory response within the tissue and which in the lungs is secreted mainly by injured epithelial alveolar cells, activates a type 2 immune response that is further responsible for impaired antiviral activity and dysregulation of neutrophils [10,19].

In the presence of an adequate immune response and virus clearance, IL-33 may play a key role in driving rapid Treg-cell-dependent restoration of respiratory tissue homoeostasis, which explains the mild or asymptomatic cases of COVID-19. In susceptible individuals with SARS-CoV-2 infection and COVID-19 pneumonia, IL-33 may induce an abnormally upregulated expression of its own receptor ST2 (also known as IL-1RL1) on Treg cells, causing an increased expression of the Th2 transcription factor GATA-binding factor 3 (GATA3), which influences the suppressive function of Treg cells. The dysregulation of GATA3+ Foxp3+ Treg cells may impair immunological tolerance, increasing the production of type 2 cytokines. Expression of ST2 can likewise be favoured by TGFβ2, which is also increased in the bronchoalveolar lavage fluid of patients with COVID-19, and might further enhance ST2 expression in innate lymphoid cells: IL-33 is the key cytokine that drives these cells to differentiate into type 2 innate lymphoid cells (ILC2s) [74,75].

The deficient levels of type I and III interferons (IFNα, IFNβ and IFNλ) that are more highly suppressed by SARS-CoV-2 than by SARS-CoV infection are related to the impaired antiviral responses and spontaneous apoptosis of dysfunctional lymphocytes [76,77,78].

Burn TN. et al., in an experimental model of haemophagocytic lymphohistiocytosis, investigated mice lacking both IFNγ and perforin (IFNγ−/−Prf1−/−) that develop a severe MAS-like disease that involves the IL-33–ST2 axis and is mediated downstream by GM-CSF-producing CD8+ T cells. The inflammatory burden in infected IFNγ−/−Prf1−/− mice is higher than in Prf1−/− mice; indeed, it is characterized by a significant increase in neutrophils and stronger upregulation of IL-1β and IL-6. Therefore, the same interaction between IL-33 and GM-CSF might occur in patients with COVID-19, initiating the cytokine storm syndrome [79].

Thus, as reported above, SARS-CoV-2 infection acts as a trigger in damaging epithelial cells in the lung causing the release of IL-33, with a consequent type 2 immune response that further leads to impaired antiviral activity, dysregulation of neutrophils and delayed viral clearance. These events favour the progression of COVID-19, with the possible extensive injury of alveolar epithelial cells, with the release of IL-33. Therefore, IL-33 acts as a protagonist in maintaining and advancing lung inflammation, in which many innate pro-inflammatory cytokines, leading to the most severe forms of COVID-19, are involved [18,40]. During COVID-19 infection, the activation of mast cells, well-expressed in nasal epithelial and lung cells, results in a hyperinflammatory syndrome through the release of inflammatory molecules, participating in a cytokine storm and, over a longer period, causing pulmonary fibrosis. The literature also reports the useful prognostic role of basophil counts for COVID-19, as a reduction is associated with a worse prognosis [35].

**Figure 3:**
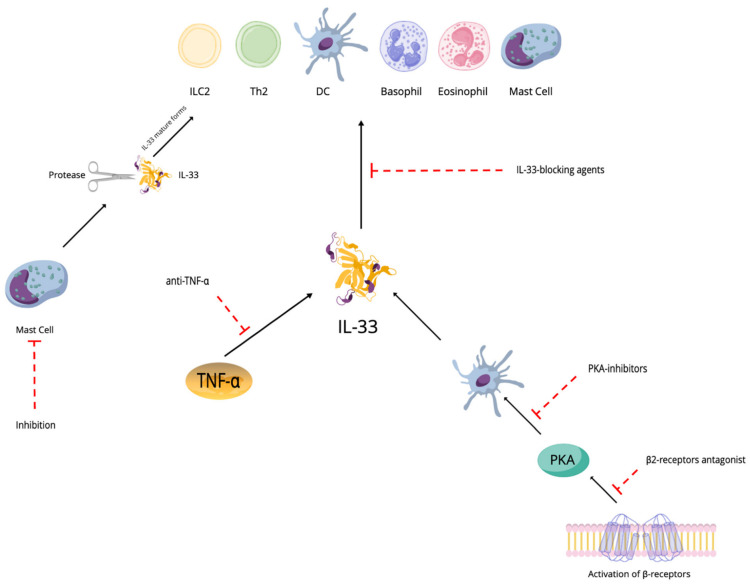
Possible therapeutic strategies.

From a therapeutic point of view (Figure 3), considering that IL-33 induces the production of pro-inflammatory cytokines from cells that express ST2 cells and hematopoietic cells including ILC2s, mast cells, Th2 cells, eosinophils, basophils and dendritic cells, the potential use of IL-33-blocking agents may be a novel therapeutic strategy to treat allergic diseases and some conditions characterized by this inflammatory profile [36,79]. Glucocorticoids usually induce suppression of the mRNA expression of pro-inflammatory mediators, but in the case of pulmonary inflammation mediated by IL-33, there may be glucocorticoid resistance [80,81]. It has been reported that serum levels of IL-33 were significantly increased in conditions such as psoriasis, psoriatic arthritis and pustular psoriasis, and related to TNF-α. Therefore, anti-TNF-α therapy may be used in IL-33-related diseases [82]. As the activation of β2-receptors and protein kinase A (PKA) might induce IL-33 mRNA expression in dendritic cells, β-receptor blockers and PKA inhibitors may also be used as IL-33-blocking agents [83,84]. In studies conducted in mice models, it has been reported that butyrate inhibits the proliferation and function of ILC2s by inhibiting intracellular GATA3 activity to suppress IL-33-mediated airway inflammation. Similar results were reported in human ILC2s [85].

Moreover, considering that mast cell proteases can cleave full-length IL-33 to a more active IL-33 domain, they can become a potential therapeutic target for IL-33-mediated allergic diseases.

Therefore, in this paper we report a broad view of the pathophysiological mechanisms and the possible therapeutic implications of IL-33, also presenting the upstream and downstream mechanisms of the activation of this interleukin, understanding how the molecular mechanisms on which it acts can be different, depending on the underlying disease mechanism and the inflammatory phenotype presented by the patient.

Further studies on IL-33 are needed to develop specific immunological treatment, with the aim of blocking the cytokine storm that is responsible for the most severe form of COVID-19, in particular in patients with comorbidities.

## Data Availability

Not applicable.
